# The Gibberellin 2-Oxidase Gene *GhGA2ox15* Positively Regulates Drought Resistance in Upland Cotton

**DOI:** 10.3390/ijms27114712

**Published:** 2026-05-23

**Authors:** Shujie Li, Mingxuan Hu, Juling Feng, Dongli Sun, Shuxun Yu, Zhen Feng

**Affiliations:** The Key Laboratory for Quality Improvement of Agricultural Products of Zhejiang Province, College of Advanced Agricultural Sciences, Zhejiang A&F University, Hangzhou 311300, China; lisj624@163.com (S.L.); hmx19990525@163.com (M.H.); 2022060037@nwafu.edu.cn (J.F.); 2019101021004@stu.zafu.edu.cn (D.S.); yushuxun@zafu.edu.cn (S.Y.)

**Keywords:** *GhGA2ox15*, cotton, drought, gibberellin 2-oxidase, reactive oxygen species (ROS)

## Abstract

Cotton is recognized as the primary source of essential natural fibers for the global textile industry, supporting its sustainability and development. However, adverse environmental conditions such as drought severely constrain cotton production; thus, developing stress-tolerant cultivars via molecular breeding is essential for maintaining yield stability. Here, a comprehensive functional dissection was conducted on *GhGA2ox15*, a gibberellin 2-oxidase gene derived from *Gossypium hirsutum* L. This gene encodes a key catabolic enzyme implicated in the deactivation of endogenous bioactive GAs and the modulation of stress adaptation. We characterized *GhGA2ox15*, a GA2ox gene from upland cotton that modulates endogenous bioactive GA levels and abiotic stress tolerance. Bioinformatics and sequence analyses confirmed that *GhGA2ox15* is a canonical C_20_-GA2ox subfamily member, with conserved DIOX_N and 2OG-FeII_Oxy domains and marked similarity to orthologs in *Arabidopsis* and rice. Tobacco subcellular localization assays indicated that *GhGA2ox15* resides in both the nucleus and the cytoplasm. In transgenic *Arabidopsis* and *Oryza sativa* lines, *GhGA2ox15* overexpression was shown to increase drought tolerance, while virus-induced gene silencing (VIGS) of *GhGA2ox15* yielded significantly compromised drought resistance. Physiological assays linked *GhGA2ox15* silencing to impaired reactive oxygen species (ROS) detoxification. The suppressed lines displayed markedly lower antioxidant enzyme activities, concomitant ROS accumulation in leaves, and attenuated transcription of drought-responsive marker genes. Our findings delineate the mechanistic role of *GhGA2ox15* in drought adaptation and highlight its potential utility in breeding drought-tolerant cotton.

## 1. Introduction

*Gossypium hirsutum* L. ranks among the world’s principal fiber crops, with extensive cultivation spanning Asia, Africa, and the Americas. Its lint constitutes a foundational input for global textile manufacturing [1,2]. In recent years, due to intensified competition for arable land, rising production costs, and frequent natural disasters caused by global climate change, the global cotton planting area has shown a fluctuating downward trend [3]. Single boll weight, plant architecture, stress resistance, and other related traits are key agronomic traits affecting cotton yield and fiber quality [4,5,6,7,8]. Therefore, the application of molecular breeding technologies to develop new cotton cultivars with robust stress tolerance is highly important for ensuring the stable development of the global cotton industry.

GAs are essential tetracyclic diterpenoid phytohormones that govern multiple facets of plant ontogeny. They potently promote seed germination, facilitate shoot and root growth, modulate floral transition, accelerate leaf expansion, and play indispensable roles in pollen maturation and fruit development [9,10,11,12,13,14]. In addition to these growth-regulating functions, GAs also participate in the modulation of plant responses to diverse abiotic stresses, thus demonstrating versatile biological activities and profound physiological importance [15,16,17,18].

Beyond directing core developmental events such as germination and vegetative expansion, GAs modulate plant responses to adverse conditions, with a well-documented influence on drought adaptation [19,20,21]. Water deficit disrupts GA homeostasis through several interdependent mechanisms. It transcriptionally suppresses key biosynthetic genes such as CPS and KS [22], thereby limiting GA production. Simultaneously, drought alters the abundance of GA receptors (e.g., GID1) and downstream DELLA proteins [23,24], attenuating GA signal perception. MYB, WRKY, NAC, and bZIP transcription factors likewise interface with GA metabolism by targeting cognate cis-motifs (G-box, P-box) within promoter regions of GA-related loci [25,26,27,28,29,30]. Additionally, post-transcriptional regulation via miRNAs [31] and upstream modulation by Ca^2+^/MAPK cascades [32,33,34] contribute to reshaping GA-mediated growth under water-limited conditions. Drought stress and salt stress, two major abiotic stresses affecting plant growth, induce distinct physiological responses in plants. Malondialdehyde (MDA), catalase (CAT), peroxidase (POD), soluble sugars (SS), superoxide dismutase (SOD), and proline (Pro) are key physiological indexes for evaluating plant stress responses and tolerance. As the primary product of plant cell plasma membrane lipid peroxidation, MDA is a typical marker of membrane system damage, whose cellular content directly reflects the degree of plasma membrane peroxidation and concomitant membrane system injury. In general, MDA levels in plants are positively correlated with the severity of abiotic stress-induced cellular damage. A previous study has demonstrated that *Arabidopsis atga3ox1/2* mutants exhibit reduced endogenous GA contents and a concomitant increase in abiotic stress tolerance [35]. In contrast, the *atga2ox* mutant accumulates higher levels of endogenous GAs and displays increased sensitivity to abiotic stress [36]. Overexpression of *GhGA2ox1* confers enhanced tolerance to drought and salinity in cotton [37].

GA2ox-mediated GA inactivation in *Arabidopsis* and rice is well documented to modulate stress adaptation. In cotton, however, the phylogenetic organization, subcellular distribution, and mechanistic roles of individual *GhGA2ox* members—particularly in shaping plant architecture and drought resilience—remain largely unexplored. This knowledge gap has severely hindered the precise application of GA pathway genes in the molecular design and breeding of cotton. To address this gap, we functionally characterized *GhGA2ox15* by generating transgenic overexpression lines in *Arabidopsis*, heterologous expression lines in rice, and VIGS lines in cotton. Drought tolerance assays demonstrated that constitutive *GhGA2ox15* overexpression enhanced drought resistance in transgenic *Arabidopsis* and rice. Conversely, cotton plants with reduced *GhGA2ox15* expression exhibited impaired drought tolerance, linked to elevated ROS levels and attenuated transcription of drought-associated genes.

## 2. Results

### 2.1. GhGA2ox15 Displays Typical Features of GA2ox Family Proteins

**Figure 1:**
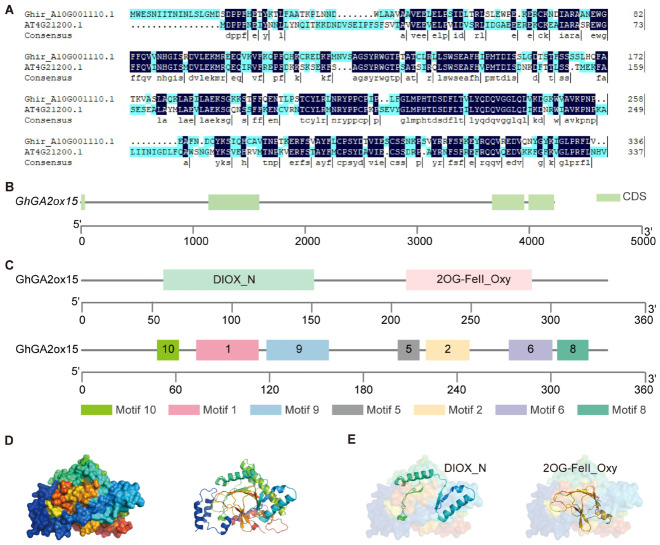
Sequence and structural analysis of GhGA2ox15: (**A**) Sequence alignment of GhGA2ox15 and AtGA2ox8 proteins. (**B**) Schematic diagram of the *GhGA2ox15* gene structure. (**C**) Conserved domain and motif composition of GhGA2ox15. (**D**) Structure of GhGA2ox15. (**E**) DIOX_N and 2OG-FeII_Oxy domain of GhGA2ox15.

GA2ox proteins play key roles in abiotic stress adaptation. For example, overexpression of *AtGA2ox8* improves drought tolerance in *Arabidopsis*, and drought-induced expression of *OsGA2ox8* restricts shoot growth to enhance stress resistance in rice. Therefore, we cloned *GhGA2ox15* from *Gossypium hirsutum* using homologous cloning [38]. Specific primers are shown in Table S2. Phylogenetic analysis classified GhGA2ox15 into the C_20_-GA2ox subfamily, with high sequence similarity to AtGA2ox8 (Figure 1A), suggesting that they may share similar protein structures and biological functions. *GhGA2ox15* exhibits a compact gene structure, consisting of a single continuous coding sequence (CDS) with no introns (Figure 1B). This structural feature is consistent with that of several reported GA2ox family genes, suggesting a potentially straightforward regulatory mode during posttranscriptional processing [39]. The GhGA2ox15 protein harbors two conserved domains, DIOX_N and 2OG-FeII_Oxy, along with seven highly conserved signature motifs (Motifs 1, 2, 5, 6, 8, 9, and 10). This high degree of evolutionary conservation provides a structural basis for its function in gibberellin oxidation (Figure 1C). Integrating AlphaFold 3 predictions with PyMOL visualization, we reconstructed the three-dimensional structure of GhGA2ox15 (Figure 1D). The structural model provides critical insights for dissecting its substrate-binding mode and enzymatic mechanism. Specifically, the DIOX_N domain serves as the primary substrate-binding module, anchoring gibberellin precursors and stabilizing the catalytic core (Figure 1E), and the 2OG-FeII_Oxy domain acts in concert with DIOX_N to form the functional catalytic unit responsible for gibberellin oxidation.

Collectively, these structural and sequence analyses confirm that *GhGA2ox15* is a canonical member of the GA2ox family, laying a solid foundation for future investigations into its catalytic activity, molecular interactions, and regulatory roles in plant gibberellin homeostasis.

### 2.2. Molecular Characterization and Expression Pattern of GhGA2ox15

**Figure 2:**
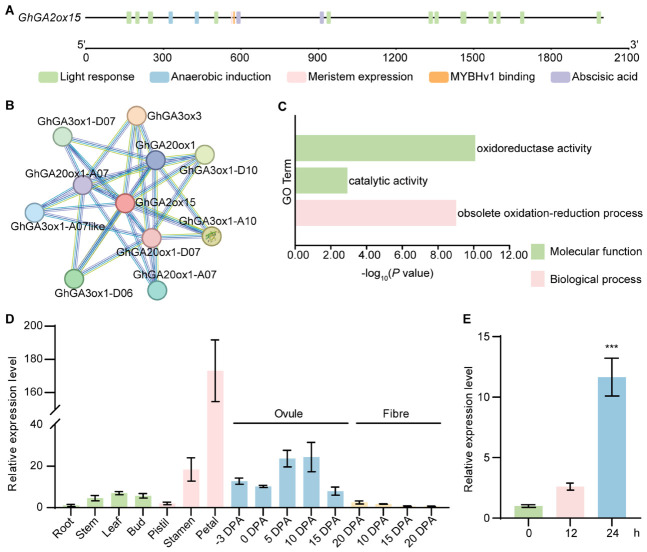
Functional prediction and expression analysis of *GhGA2ox15*: (**A**) Cis-regulatory element analysis of the *GhGA2ox15* promoter. (**B**) Prediction of the protein interaction network of GhGA2ox15. (**C**) GO functional enrichment analysis of GhGA2ox15-interacting proteins. (**D**) Organ-specific expression pattern of *GhGA2ox15*. Green bars represent vegetative tissues (root, stem, leaf, bud); pink bars represent floral organs (pistil, stamen, petal); blue bars represent developing ovules; orange bars represent developing fibers. (**E**) Transcript levels of *GhGA2ox15* after GA_3_ treatment. Data are presented as the mean ± standard error (SE) of three biological replicates. *** *p* < 0.001 (Student’s *t*-test).

Promoter analysis of the 2000 bp upstream regulatory region of *GhGA2ox15* revealed several cis-regulatory elements linked to light responsiveness, anaerobic induction, meristem activity, MYBHV1 binding, and ABA signaling (Figure 2A; Table S2). These features suggest that *GhGA2ox15* transcription integrates both developmental and environmental inputs. To identify potential interaction partners, a protein-protein interaction (PPI) network was built using the STRING database, with *GhGA2ox15* as the central node (Figure 2B); the results revealed potential interactions between GhGA2ox15 and ten proteins: GhGA3ox1-A10, GhGA3ox3, GhGA3ox1-A07, GhGA20ox1, GhGA3ox1-D06, GhGA3ox1-like, GhGA3ox1-D07, GhGA3ox1-D10, GhGA20ox1-D07, and GhGA20ox1-A07. These proteins belong to the GA3ox and GA20ox families and function as core catalysts in gibberellin biosynthesis, converting precursors into bioactive forms that drive plant ontogeny. Gene Ontology (GO) enrichment analysis of GhGA2ox15-interacting proteins revealed that these interactors were enriched primarily in catalytic and oxidoreductase activities with high specificity (Figure 2C), suggesting that GhGA2ox15 may be involved in hormone metabolism (e.g., GA inactivation) and redox homeostasis via enzyme interactions, providing clues for studying its molecular mechanism. Furthermore, quantitative real-time PCR (qRT-PCR) analysis (Figure 2D) demonstrated that *GhGA2ox15* is predominantly expressed in reproductive organs, especially petals and stamens. *GhGA2ox15* transcript levels were relatively high during early fiber development (−3 to 10 DPA) but decreased markedly at later stages (15–20 DPA), suggesting a potential role in fiber initiation and early elongation. Moreover, exogenous GA_3_ treatment significantly induced *GhGA2ox15* expression at 24 h (Figure 2E), indicating that this gene is positively feedback-regulated by bioactive gibberellins.

Our results indicate that GhGA2ox15 interacts with core GA biosynthetic enzymes (GA3ox and GA20ox family members), shows enriched expression in reproductive organs and during early fiber initiation, and responds positively to GA_3_, implying a central function in GA homeostasis and stress adaptation in cotton.

### 2.3. GhGA2ox15 Exerts Its Functions in Both the Nucleus and Cytoplasm

**Figure 3:**
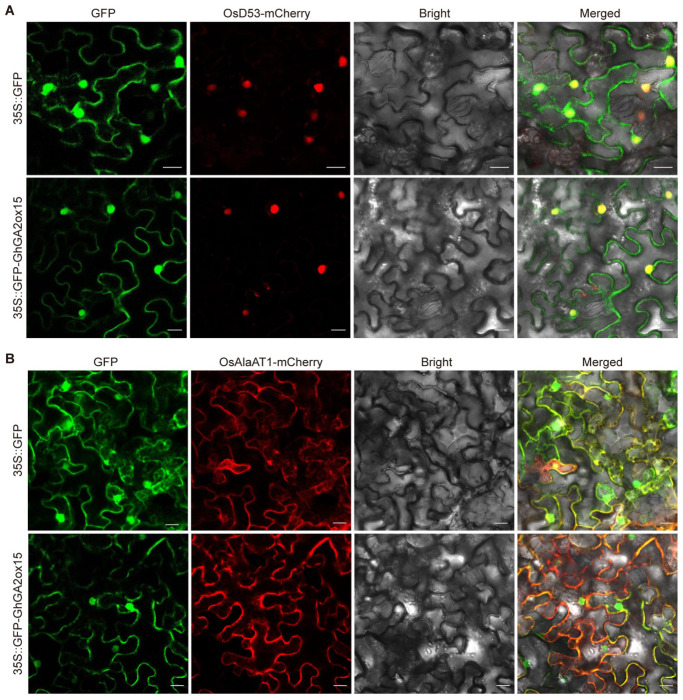
GhGA2ox15 functions in the nucleus and cytoplasm: (**A**) Co-localization of 35S::GFP-GhGA2ox15 with the nuclear marker OsD53-mCherry. (**B**) Co-localization of 35S::GFP-GhGA2ox15 with the cytoplasmic marker OsAlaAT1-mCherry. The 35S::GFP empty vector was used as a control. OsD53 and OsAlaAT1 are well-established nuclear and cytoplasmic markers, respectively, validated for use in tobacco transient expression systems. Bars = 20 μm.

On the basis of predictions using the online database WoLF PSORT, we hypothesized that GhGA2ox15 functions in both the cytoplasm and the nucleus. We cloned the *GhGA2ox15* coding sequence and successfully constructed the recombinant vector pCAMBIA-GFP-*GhGA2ox15*. We subsequently observed the subcellular localization of this gene in tobacco leaves. A 35S::GFP empty vector control was included to distinguish specific localization signals. The nuclear marker *OsD53* [40] and cytoplasmic marker *OsAlaAT1* [41] were co-expressed as compartment references in tobacco epidermal cells. Notably, this dual localization pattern in the cytoplasm and nucleus is highly relevant to stress response regulation, as it enables GhGA2ox15 to simultaneously participate in cytoplasmic GA metabolic regulation and nuclear transcription-related processes involved in stress adaptation. The results revealed that GFP-GhGA2ox15 was colocalized with both a nuclear localization marker and a cytoplasmic localization marker (Figure 3), findings that are consistent with the subcellular localization profiles of *PavGA2ox-2L* [42] from sweet cherry, *OsGA2ox5* [43] and *OsGA2ox6* [44] from rice, and *AtGA2ox4* [45] from *Arabidopsis*. This consistency further supports the potential role of *GhGA2ox15* in drought tolerance, as the conserved subcellular localization among these homologous *GA2ox* genes implies that conserved functional pathways mediate stress resistance through GA deactivation in both compartments.

### 2.4. GhGA2ox15 Overexpression Improves Drought Tolerance in Arabidopsis

**Figure 4:**
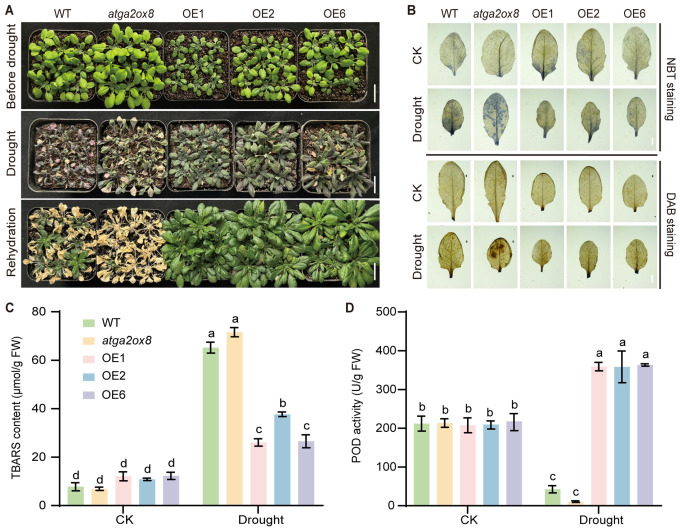
Ectopic expression of *GhGA2ox15* improves drought tolerance in *Arabidopsis*: (**A**) Plant phenotypes under normal growth, drought stress, and rehydration conditions. Bars = 2 cm. (**B**) Phenotypes of *Arabidopsis* leaves after nitroblue tetrazolium (NBT) and 3,3′-diaminobenzidine (DAB) staining. Bars = 0.4 mm. (**C**,**D**) Analysis of the thiobarbituric acid reactive substances (TBARS) content (**C**) and POD activity (**D**) in the leaves of WT and *GhGA2ox15*-overexpressing lines after drought stress treatment. Data are shown as the mean ± SE of three independent biological replicates. Columns marked with different letters represent significant differences (*p* < 0.05, one-way ANOVA followed by Tukey’s test).

Previous studies have shown that members of the GA2ox gene family are involved in the regulation of plant responses to various biotic and abiotic stresses. For instance, the overexpression of upland cotton *GhGA2ox1* confers enhanced tolerance to salinity and water deficit [37], and preliminary research on *Arabidopsis AtGA2ox8* suggests that its overexpression improves drought resistance [46]. To investigate the role of *GhGA2ox15* in drought tolerance, *GhGA2ox15* was constitutively expressed in wild-type (WT) *Arabidopsis*. The T_3_ overexpression lines displayed obvious growth inhibition and reduced plant height compared with WT (Figure S1A–C). Three individual T_3_ homozygous overexpression lines (OE1, OE2, and OE6) were used for further characterization. To assess the role of *GhGA2ox15* in drought tolerance, water was withheld from seedlings. After 15 days of water deprivation, relative to WT and *atga2ox8* mutants, the OE lines exhibited markedly reduced leaf wrinkling and wilting, with higher survival rates post-rehydration (Figure 4A). We further measured fresh and dry weights of above-ground and underground tissues under drought treatment, and the biomass performance also supported that *GhGA2ox15* overexpression alleviated drought-induced growth inhibition.

To quantify the phenotypic differences, we measured physiological parameters of drought-stressed and non-stressed *Arabidopsis* plants. To assess leaf ROS accumulation, nitroblue tetrazolium (NBT) and 3,3′-diaminobenzidine (DAB) staining were performed using untreated control (CK) and drought-stressed plants (Figure 4B). Compared with that in the CK group, the NBT staining intensity was greater in the drought-stressed plants, with the most intense staining observed in the *atga2ox8* mutants, followed by the WT plants; the OE lines presented relatively faint blue coloration. DAB staining yielded similar results. These findings indicate that the accumulation of O^2−^, H_2_O_2_, and other ROS was markedly elevated in WT and *atga2ox8* plants, leading to more severe oxidative damage and greater drought sensitivity. In contrast, the OE lines possessed superior antioxidant capacity to maintain lower ROS levels. We further measured thiobarbituric acid reactive substances (TBARS) content (Figure 4C) and POD activity (Figure 4D) to verify these findings. Under normal conditions, TBARS accumulation was comparable across all the lines, with no significant differences. After drought, TBARS levels increased significantly in all lines, with notably higher accumulation in the WT and *atga2ox8* lines than in the OE lines, indicating more severe oxidative stress in the former. Under well-watered conditions, POD activity was comparable across all lines. Drought impaired the antioxidant system of WT and *atga2ox8* plants, reducing their POD activity, whereas OE1, OE2, and OE6 plants presented higher POD activity after drought. Together with the results of the NBT and DAB staining, these data confirm that the antioxidant capacity of the OE lines was stronger than that of the other lines and that these lines maintained lower TBARS and ROS levels as well as higher POD activity under drought stress. In conclusion, *GhGA2ox15* overexpression enhanced the capacity of *Arabidopsis* plants to respond to drought, reduced ROS accumulation, mitigated oxidative damage, and thereby improved drought resistance in the transgenic lines.

### 2.5. GhGA2ox15 Overexpression Improves Drought Tolerance in Rice

GA2ox family members are recognized as key modulators of rice stress adaptation. Specific mutant alleles of *osga2ox6* confer improved resilience to cold, salinity, drought, and heat [44]. Additionally, *OsGA2ox8* overexpression elevated osmoprotectants and antioxidants under osmotic stress, thus enhancing osmotic stress tolerance [47].

**Figure 5:**
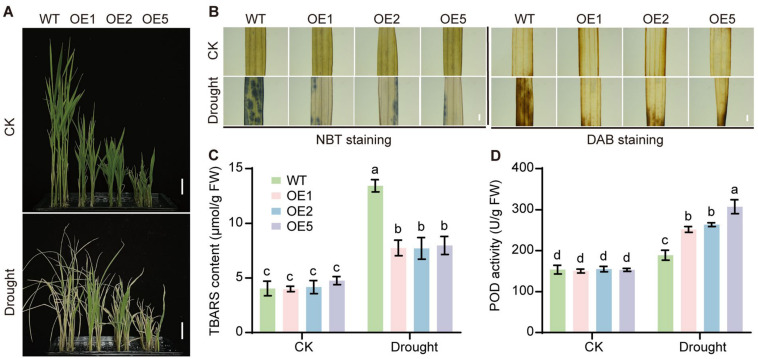
*GhGA2ox15* overexpression in rice enhances plant drought resistance: (**A**) Phenotypes of WT and transgenic rice under normal growth and PEG-simulated conditions. Bars = 2 cm. (**B**) NBT and DAB staining of rice leaves to detect O^2−^ and H_2_O_2_ accumulation, respectively. Bars = 0.4 mm. (**C**,**D**) Analysis of the TBARS content (**C**) and POD activity (**D**) in the leaves of WT and *GhGA2ox15*-overexpressing lines after PEG-simulated drought stress treatment. Data are presented as the mean ± SE of three independent biological replicates. Columns marked with different letters represent significant differences (*p* < 0.05, one-way ANOVA followed by Tukey’s test).

Building on these conserved functions in stress responses, we generated three independent homozygous *GhGA2ox15*-overexpressing rice lines (OE1, OE2, and OE5), validated by elevated transcript levels and reduced plant height (Figure S2), and subjected them to simulated drought assays. After 14 days of culture in 1/2 MS liquid medium, rice seedlings were treated with 10% PEG 6000 for 5 days to simulate drought stress. Compared to overexpression plants, WT plants displayed markedly more severe leaf wilting (Figure 5A). We subsequently measured a series of physiological indices. NBT and DAB staining (Figure 5B) showed increased ROS signals in all genotypes under PEG-induced water stress, with significantly stronger intensity in WT leaves than in OE lines. This indicates reduced peroxide accumulation in the transgenic plants. We quantified TBARS content (Figure 5C) and POD activity (Figure 5D). Under normal conditions, TBARS content and POD activity did not differ significantly across lines. Following drought treatment, both TBARS content and POD activity increased in all plants; however, WT plants accumulated substantially more TBARS, while OE lines exhibited significantly higher POD activity than the WT controls.

Overall, *GhGA2ox15* overexpression in rice significantly increased the antioxidant capacity under drought stress, reduced ROS accumulation, alleviated oxidative damage in leaves, and increased drought tolerance in the transgenic lines.

### 2.6. GhGA2ox15 Silencing Impairs ROS Scavenging and Attenuates Stress-Responsive Gene Expression Under Drought Stress in Cotton

**Figure 6:**
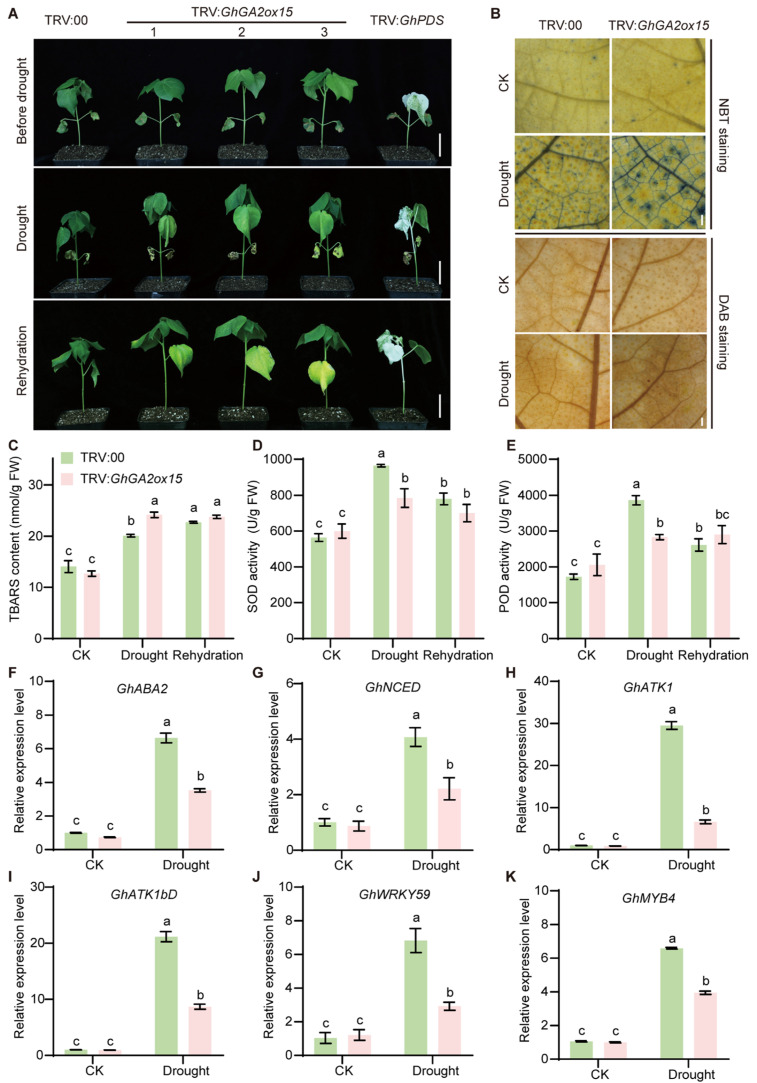
*GhGA2ox15* silencing reduces the drought resistance of cotton: (**A**) Representative phenotypes of *GhGA2ox15*-silenced cotton plants under normal growth, drought stress, and rehydration conditions. Bars = 5 cm. (**B**) Leaf phenotypes of cotton after NBT and DAB staining. Bars = 100 μm. (**C**–**E**) Quantification of TBARS content (**C**) and SOD (**D**) and POD (**E**) activities in leaves of control and *GhGA2ox15*-silenced plants under normal growth, drought stress, and rehydration conditions. (**F**–**K**) Relative expression levels of *GhABA2*, *GhNCED*, *GhATK1*, *GhATK1bD*, *GhWRKY59*, and *GhMYB4* in cotton. Data are presented as the mean ± SE of three independent biological replicates. Columns marked with different letters represent significant differences (*p* < 0.05, one-way ANOVA followed by Tukey’s test).

Building on the functional evidence obtained from heterologous overexpression in *Arabidopsis* and rice, we herein employed VIGS to downregulate the expression of *GhGA2ox15* (Figure S3B) and evaluated the drought tolerance of silenced cotton plants. Following 15 days of water withholding, TRV:*GhGA2ox15*-silenced plants exhibited more pronounced leaf wilting relative to TRV:00 control plants (Figure 6A). Key physiological indices were measured to explore the underlying mechanism. DAB and NBT staining (Figure 6B) revealed intensified signals in drought-stressed plants, with markedly stronger staining in TRV:*GhGA2ox15* plants, indicating excessive ROS accumulation. Under normal conditions, the TBARS content did not differ between the lines; drought markedly increased the TBARS content, with greater accumulation in TRV:*GhGA2ox15* plants. Upon drought stress, SOD and POD activities declined substantially in TRV:*GhGA2ox15* lines relative to controls (Figure 6C–E). Consistent with these findings, CAT activity, another key ROS-scavenging enzyme, was also significantly lower in TRV:*GhGA2ox15* plants than in TRV:00 controls under drought conditions (Figure S4), further confirming impaired antioxidant capacity in silenced plants. To elucidate the underlying molecular mechanisms, qRT-PCR assays were performed (Figure 6F–K). After drought treatment, transcript levels of the key ABA biosynthetic genes *GhABA2* [48] and *GhNCED* [49] were significantly upregulated in TRV:00 control plants compared with TRV:*GhGA2ox15*-silenced lines. Similarly, drought stress upregulated the K^+^ channel regulatory genes *GhATK1* [50] and *GhAKT1bD* [51]. This induction was markedly more pronounced in TRV:00 plants than in silenced plants; in controls, transcript levels were increased more than 20-fold relative to pre-stress values. The drought-responsive transcription factors *GhWRKY59* [52] and *GhMYB4* [53] were also significantly upregulated after drought stress. However, this induction was markedly repressed in TRV:*GhGA2ox15* plants relative to TRV:00 controls. The reduced expression of these drought-responsive genes in *GhGA2ox15*-silenced plants likely represents a key factor underlying their compromised drought tolerance. Notably, while *GhGA2ox15* directly modulates drought tolerance through regulating ROS homeostasis and stress-related gene expression, its silencing also alters plant growth (Figure S3C–E). Such GA-mediated growth changes may exert pleiotropic effects and indirectly affect drought tolerance.

## 3. Discussion

Gibberellin 2-oxidases are central regulators of gibberellin homeostasis, acting as key coordinators of plant growth and stress adaptation. This research systematically characterized *GhGA2ox15* from *Gossypium hirsutum* and explored its regulatory role in drought stress responses. Our results confirm that *GhGA2ox15* belongs to the canonical C_20_-GA2ox family, and its conserved structural features enable it to function as a positive regulator of drought tolerance. This regulatory role is mediated by modulating ABA signaling-related gene expression, enhancing antioxidant defense capability, and maintaining ROS homeostasis. Collectively, these findings enrich our understanding of GA2ox-mediated drought adaptation in cotton and provide a valuable molecular target for breeding drought-tolerant cotton varieties.

As a typical C_20_-GA2ox member, *GhGA2ox15* shares conserved domain architecture and phylogenetic affinity with functionally validated GA2ox homologs. Phenotypic evidence from multiple genetic materials firmly supports the roles of *GhGA2ox15* in GA metabolism and drought regulation. Heterologous overexpression of *GhGA2ox15* in *Arabidopsis* and rice significantly reduced plant height, whereas TRV:*GhGA2ox15* in cotton led to a noticeable increase in plant height. Such phenotypic alterations are highly consistent with the canonical function of the GA2ox family in negatively regulating plant height and mediating gibberellin catabolism. This growth-related pleiotropy may indirectly affect drought performance, in addition to the intrinsic role of *GhGA2ox15* in regulating drought stress tolerance. More importantly, consistent drought phenotypic results clearly demonstrate the core function of *GhGA2ox15* in drought responses: overexpression lines exhibited markedly enhanced drought tolerance, while silenced lines displayed elevated drought sensitivity. Previous studies have shown that GA2ox genes play crucial roles in drought responses [37,47]. For example, overexpression of *AtGA2ox8* enhances drought tolerance by modulating endogenous GA accumulation [46], while several *AtGA2ox* isoforms are widely induced by multiple abiotic stresses [54]. The gain- and loss-of-function phenotypes of *GhGA2ox15* in cotton confirm it as a positive regulator of drought tolerance. Additionally, a genome-wide study in cotton reported that *GhGA2ox1* is significantly upregulated under salt stress [55], consistent with the involvement of GA2ox family members in abiotic stress responses.

To dissect the molecular mechanisms underlying this improved drought tolerance, we profiled the expression of core stress-responsive genes. In *GhGA2ox15*-silenced cotton lines, the expression levels of *GhABA2* [48] and *GhNCED* [49] (ABA synthesis genes), *GhATK1* [50] and *GhAKT1bD* [51] (K^+^ channel protein regulatory genes), and *GhWRKY59* and *GhMYB4* (drought-responsive transcription factors) were all markedly lower than those in the control group. Across these genes, *GhABA2* and *GhNCED* are homologous to *AtABA2* and *AtNCED*, which are essential for plant drought tolerance; as transcription factors, *GhWRKY59* [52] and *GhMYB4* [53] play crucial roles in regulating the expression of drought-responsive genes. Thus, *GhGA2ox15* confers drought tolerance partly via the ABA-dependent pathway. Additionally, similar regulation occurs in rice: *OsGA2ox8* enhances ABA synthesis gene expression and osmotic tolerance, while *OsGA2ox9* deficiency reduces ABA signaling [47,56]. Importantly, both studies have demonstrated that GA2ox significantly reduces bioactive GA levels through hydroxylation. Mechanistically, GA2ox-mediated GA reduction stabilizes DELLA proteins, which interact with ABI4/ABI5 to activate NCED expression [57]. This conserved regulatory module has been well documented in *Arabidopsis* and rice, but its functional conservation in cotton remains to be fully elucidated. Our finding that *GhGA2ox15* silencing significantly attenuated the drought-induced upregulation of *GhNCED* provides direct preliminary evidence for the existence of this module in cotton.

Physiological indices further validated that *GhGA2ox15* maintains cellular homeostasis under drought stress. Overexpression of *GhGA2ox15* reduced TBARS accumulation in *Arabidopsis* and rice, whereas silencing increased TBARS levels in cotton, indicating alleviation of oxidative damage. Meanwhile, POD and SOD activities declined in silenced cotton but were markedly elevated in overexpression lines. These physiological changes correspond well with the enhanced drought resistance. Notably, while *GhGA2ox15* directly modulates drought tolerance through regulating ROS homeostasis and stress-related gene expression, its silencing also alters plant growth architecture. Such GA-mediated growth changes may exert pleiotropic effects and indirectly affect drought tolerance, which represents another important aspect that requires further investigation in future studies.

Despite these consistent findings, several limitations remain. First, we did not characterize the enzymatic activity or quantify endogenous GA levels, leaving the regulatory relationship between *GhGA2ox15*, GA metabolism, and ROS homeostasis correlative. In addition, DAB and NBT staining only provide qualitative ROS observation; further quantitative ROS measurement will be adopted in future research. Second, the upstream regulators and direct downstream targets of *GhGA2ox15* are unclear, and the GA-ABA crosstalk requires further dissection. Third, phenotypic evaluations were performed only at the seedling stage; field drought adaptability needs validation. These limitations do not affect our main conclusions but provide directions for future mechanistic studies. Fourth, fewer quantitative physiological drought indicators were detected in this study. More indexes, such as leaf water potential, stomatal conductance, and transpiration rate under standardized drought conditions, will be supplemented in future studies to improve phenotypic evaluation accuracy.

Collectively, *GhGA2ox15* positively regulates drought tolerance by modulating stress-associated gene expression and enhancing antioxidant capacity to maintain ROS homeostasis. This study provides a candidate gene for molecular breeding and extends our understanding of GA regulatory networks in cotton drought adaptation. Future work should identify upstream regulators and downstream targets of *GhGA2ox15*, clarify its role in ABA signaling, and perform enzymatic assays and GA quantification. From an applied perspective, fine-tuning *GhGA2ox15* expression may achieve moderate dwarfing and improved drought tolerance, facilitating the breeding of high-yield, drought-resistant cotton varieties.

## 4. Materials and Methods

### 4.1. Plant Materials

All *Arabidopsis* lines used were of the Columbia (Col-0) background. WT seeds were lab-maintained; the *atga2ox8* T-DNA insertion mutant (WiscDsLox263B11) was sourced from TAIR-ABRC (https://www.arabidopsis.org/). *Arabidopsis* was grown in a growth chamber under controlled conditions (23 °C/21 °C, 16 h/8 h light/dark cycle, 150 μmol m^−2^ s^−1^). This study used the upland cotton standard line ‘TM-1’ (lab-preserved). Upland cotton was cultivated either in a growth chamber under a photosynthetic photon flux density of 500 μmol·m^−2^·s^−1^ (16 h light/8 h dark photoperiod) or in the Crop Garden of Donghu Campus, Zhejiang A&F University (30°20′ N, 119°34′ E). Growth chamber conditions: 28 °C/25 °C, 16 h/8 h light/dark. *Nicotiana benthamiana* tobacco plants were grown under the same environmental conditions as *Arabidopsis*, with the exception of light intensity, which was maintained at 300 μmol m^−2^ s^−1^. Zhonghua 11 rice was germinated in a growth chamber under the same conditions as cotton.

### 4.2. Bioinformatics Analysis of GhGA2ox15

Genome and GFF3 annotation files for *Gossypium hirsutum* cv. TM-1 (HAU1.1 assembly) were acquired from CottonGen (https://www.cottongen.org, accessed on 20 October 2020) [58]. Corresponding files for rice were retrieved from Phytozome (https://phytozome-next.jgi.doe.gov/, accessed on 5 February 2023). A 2000 bp *GhGA2ox15* promoter fragment was retrieved from HAU1.1 and analyzed for cis-elements via PlantCARE (http://bioinformatics.psb.ugent.be/webtools/plantcare/html/, accessed on 23 March 2023). PPI partners of GhGA2ox15 were predicted via the STRING database (https://cn.string-db.org/, accessed on 16 April 2023). GO enrichment analysis of the interacting proteins was subsequently conducted with TBtools-II (v2.435) [59]. First, the conserved domains of the GhGA2ox15 protein were predicted using SMART (https://web.expasy.org/protparam/, accessed on 25 May 2023). The protein sequence was submitted to AlphaFold 3.0 (https://alphafoldserver.com/, accessed on 30 December 2025) to obtain the predicted three-dimensional structure in PDB format. The protein structure and conserved domains were then visualized using PyMOL (v3.1.6.1).

### 4.3. Subcellular Localization Analysis

Subcellular localization of GhGA2ox15 was predicted using the WoLF PSORT online tool (https://wolfpsort.hgc.jp/, accessed on 10 March 2022). To determine the subcellular distribution of *GhGA2ox15* in plant cells, we performed transient expression assays using tobacco (*Nicotiana benthamiana*). The 1002-bp coding sequence of *GhGA2ox15* (*Ghi_A10G01391*; GenBank accession: XM_016855697.2) was amplified from upland cotton cultivar ‘TM-1’ and cloned into the pCAMBIA1305-GFP vector. The resulting construct, under the control of the CaMV 35S promoter, produces a GhGA2ox15-GFP fusion protein. The validated construct was used to transform *Agrobacterium tumefaciens* strain GV3101. For co-localization assays, OsD53-mCherry was used as a nuclear localization marker and OsAlaAT1-mCherry as a cytoplasmic localization marker; both markers have been well-validated and widely applied in tobacco transient expression systems in previous studies [33,60]. The *GhGA2ox15*-GFP construct was co-infiltrated with the nuclear marker 35S-*OsD53*-mCherry and the cytoplasmic marker 35S-*OsAlaAT1*-mCherry into leaves of 3–4-week-old tobacco plants. Additionally, the empty pCAMBIA1305-GFP vector (without the *GhGA2ox15* coding sequence) was separately infiltrated as a negative control to eliminate background fluorescence interference; this control produces free GFP that diffuses throughout the nucleus and cytoplasm, allowing for a clear distinction from the fusion protein’s specific localization. At 48 h post-infiltration, GFP fluorescence was imaged with confocal laser scanning microscopy (LSM 880; Zeiss, Oberkochen, Germany). Co-localization was assessed by merging the GFP (green) and mCherry (red) channels, and yellow fluorescence in merged images indicated overlap of GhGA2ox15-GFP with the respective marker. All subcellular localization experiments were performed with three biological replicates and three technical replicates to ensure experimental reproducibility.

### 4.4. Expression Analysis of GhGA2ox15

Expression of *GhGA2ox15* was profiled across major fiber developmental stages—initiation (−3 to 0 DPA), elongation (1–15 DPA), and secondary wall synthesis (20–25 DPA)—and in various cotton organs, as previously described [61]. Spatial expression was also examined in different tissues, including roots, stems, leaves, shoot apices, petals, pistils, and stamens. Total RNA was isolated using an RNA Prep Pure Plant Kit. First-strand cDNA was synthesized from 1 μg of total RNA using a reverse transcription kit according to the manufacturer’s instructions. The qRT-PCR was performed with SYBR Green PCR Master Mix on a real-time PCR system. The cotton *GhACT4* gene was used as an internal reference gene to normalize the expression levels of *GhGA2ox15* [62]. Relative gene expression was calculated using the 2^−ΔΔCT^ method [63]. All reactions were performed with three biological replicates and three technical replicates.

### 4.5. Identification and Phenotypic Analysis of Transgenic Arabidopsis

To further characterize the biological functions of *GhGA2ox15* in planta, the full-length coding sequence of *GhGA2ox15* was cloned into the pBI121 expression vector via homologous recombination using specific amplification primers. The recombinant plasmid was transformed into *Agrobacterium tumefaciens* GV3101 by the freeze–thaw method. Stable transformation of *Arabidopsis* was performed via the floral dip method. Transgenic seeds were germinated on 1/2 MS medium containing 50 mg/L kanamycin for 7–10 days, and resistant seedlings were transplanted into a 3:1:1 mixture of soil, vermiculite, and perlite for subsequent phenotypic observation.

Drought tolerance assays were conducted among WT, mutant, and *GhGA2ox15*-overexpressing *Arabidopsis* lines. All phenotypic assessments and drought treatments were performed with at least three independent biological replicates, and each sample was detected with three technical replicates to guarantee experimental accuracy and reproducibility. For drought treatment, seeds were directly sown in small pots and cultivated for three weeks; then, water withholding was applied until obvious phenotypic differences emerged. Plant phenotypes were photographed, and leaf samples were collected for subsequent determination of physiological indices.

### 4.6. Assessing Drought Tolerance in Cotton Using a VIGS-Based Approach

A fragment of *GhGA2ox15* was cloned into the VIGS vector pTRV2 to explore its function in cotton [64]. The recombinant construct was verified by Sanger sequencing prior to genetic transformation. After confirmation, the verified plasmid was introduced into Agrobacterium tumefaciens strain GV3101 for subsequent VIGS experiments. Cotton seedlings with uniform growth status and intact cotyledons were selected for infiltration. The prepared bacterial solution was slowly injected into cotyledons using a syringe until more than 90% of the leaf area was infiltrated. The positive control plants developed an albino phenotype at 7–10 days post-inoculation. Samples were collected for RNA extraction to assess gene silencing efficiency via qRT-PCR. All VIGS treatments, phenotypic observations, and physiological measurements were performed with at least three independent biological replicates, and each sample was analyzed with three technical replicates to ensure experimental reproducibility. Three weeks post-inoculation, watering was withheld. After 15 days of drought treatment, when distinct drought-responsive phenotypes were observed, the TBARS content, POD activity, and SOD activity were determined. The same physiological indices were measured again three days after rewatering.

### 4.7. NBT and DAB Staining for ROS Detection

ROS accumulation in drought-treated and control leaves of *Arabidopsis*, rice, and cotton was assessed using DAB and NBT staining. For DAB staining, leaf segments were vacuum-infiltrated in DAB solution at 0.06–0.08 MPa for 30 min, followed by incubation in the dark at room temperature for 2 h. For NBT staining, leaf segments were vacuum-infiltrated in NBT solution under the same pressure conditions for 30 min, followed by dark incubation at room temperature for 2 h. After incubation, chlorophyll was removed using anhydrous ethanol, and ROS accumulation was observed and photographed under a stereomicroscope. Reddish-brown precipitates indicated H_2_O_2_ accumulation, while blue precipitates indicated O^2−^ accumulation. All staining assays were performed with three biological replicates and three technical replicates to ensure experimental reproducibility.

### 4.8. Determination of TBARS Content and POD and SOD Activities

Leaf tissue (0.5 g) was homogenized in 50 mM phosphate buffer (pH 7.8) containing polyvinylpyrrolidone (PVP). The homogenate was centrifuged at 10,000 rpm for 20 min at 4 °C, and the supernatant was retained as the crude enzyme extract. TBARS content was assayed using 0.6% thiobarbituric acid (TBA), and absorbance was recorded at 532 nm after a boiling water bath reaction. Due to the interference of endogenous phenolic and carbohydrate compounds in plant tissues, the TBA method cannot specifically quantify malondialdehyde (MDA); it actually detects TBARS. Thus, TBARS content was used to evaluate membrane lipid peroxidation in this study [65]. Peroxidase (POD) activity was measured via the guaiacol method in a reaction system containing phosphate buffer and H_2_O_2_, with detection at 470 nm. Superoxide dismutase (SOD) activity was determined by the NBT photoreduction method using methionine and riboflavin, and the absorbance was measured at 510 nm. All physiological measurements were performed with at least three independent biological replicates, and each sample was assayed with three technical replicates to ensure data reliability and reproducibility. All major phenotypic experiments were independently repeated three times, yielding consistent results. Representative data from one experiment or pooled data from three replicates are shown in the figures.

## 5. Conclusions

In summary, *GhGA2ox15* was identified as a canonical C_20_-GA2ox family gene whose conserved structural domains are required for gibberellin oxidation. It possesses the signature DIOX_N and 2OG-FeII_Oxy domains characteristic of this family. The cloned *GhGA2ox15* gene encoded a gibberellin oxidase that was highly expressed in floral organs and responded to exogenous gibberellin induction, and the protein resided in the nucleus and cytoplasm. Notably, we found that *GhGA2ox15* overexpression enhanced drought resistance in *Arabidopsis* and rice, whereas the silencing of this gene severely compromised drought tolerance in *G. hirsutum*, accompanied by reduced antioxidant enzyme activity, excessive ROS accumulation, and downregulated drought-responsive gene expression. Specifically, the results indicated that *GhGA2ox15* mediates plant drought tolerance by modulating endogenous gibberellin abundance and preserving ROS homeostasis. This study identifies *GhGA2ox15* as a crucial drought response regulator in cotton, offering a novel target for stress-resistance breeding. Meanwhile, this study enriches the understanding of the stress-resistant function of the C_20_-GA2ox family, improves the molecular network where gibberellin metabolism and reactive oxygen species homeostasis synergistically regulate drought resistance, and its cross-species conservation provides candidate genes and theoretical references for the molecular breeding of drought tolerance in cotton and other crops. In the future, further investigation into its regulatory network and biochemical functions is needed to clarify the molecular mechanisms underlying its role in drought tolerance.

## Data Availability

The original contributions presented in this study are included in the article/Supplementary Materials. Further inquiries can be directed to the corresponding author.

## Supplementary Materials

The following supporting information can be downloaded at: https://www.mdpi.com/article/10.3390/ijms27114712/s1.
