# Supplementary material for: The Gibberellin 2-Oxidase Gene GhGA2ox15 Positively Regulates Drought Resistance in Upland Cotton
Source: Int J Mol Sci. 2026 May 23;27(11):4712. doi: 10.3390/ijms27114712 (PMC13256557; doi:10.3390/ijms27114712)
Supplement: Supplementary file 1 [file ijms-27-04712-s001.zip › Supplement Tables.pdf]

Supplementary Tables for

Gibberellin 2-oxidase Gene *GhGA2ox15* Positively Regulates Drought Resistance in Upland Cotton

Supplementary Table S1. Primer pairs used in this study

Supplementary Table S2. Analysis of *GhGA2ox15* promoter

Supplementary Table S1 Primer pairs used in this study

Usage	Primer Name	Sequence (5′ - 3′)
Construct for clone	TA-GhGA2ox15-F	ATGGATTCGGACCCACCATTC
	TA-GhGA2ox15-R	TTATACAAGAAACCTTGGAAGCCC
Construct for overexpression	pBI121- GhGA2ox15-F	ACGGGGGACTCTAGAATGGATTCG GACCCACCATTCC
	pBI121- GhGA2ox15-R	GGGAAATTCGAGCTCTTATACAAGA AACCTTGGAAGCCC
Construct for VIGS analysis	TRV- GhGA2ox15-F	GTGAGTAAGGTTACCGAATTCCCGG TTGAACAGATACCCACC
	TRV- GhGA2ox15-R	GAGCTCGGTACCGGATCCCAAGAA ACCTTGGAAGCCC
Construct for localization analysis	1305-35S-GhGA2ox15-GFP-F	GCCCAGATCAACTAGTATGGATTCGG ACCCACCATTC
	1305-35S-GhGA2ox15-GFP-R	TCGAGACGTCTCTAGATTATACAAGA AACCTTGGAAGCCC
RT-qPCR	qPCR-GhGA2ox15-F	CAGTTCGCTACAAAAGTTGCCAG
	qPCR-GhGA2ox15-R	GGATCGGACATGGTGGGTATCTG
	qPCR-GhABA2-F	TCACTGTCCAGCCAAAGATTA
	qPCR-GhABA2-R	GCACATCAACAATGCAGACT
	qPCR-GhNCED-F	TGACACCTCCCGACTCTATT
	qPCR-GhNCED-R	CGTGTGGACTTACCTGTCTTC
	qPCR-GhMYB4-F	TTAGGCAATCGGTGGTCG
	qPCR-GhMYB4-R	AATTATGATGTGGGCGGTTG
	qPCR-GhWRKY59-F	AGATCAGATGACGACCCGAC
	qPCR-GhWRKY59-R	TGTTGCTGTTGGTATGGCTC
	qPCR-GhAKT1-F	ACAATGGGGCAAACATCAAT
	qPCR-GhAKT1-R	CGCCATAACGAACGATTTCT

Usage	Primer Name	Sequence (5′ - 3′)
	qPCR-GhAKT1bD-F	GTTGCGTTCGTATGGCTTGTT
	qPCR-GhAKT1bD-R	CCAGGATCACGGTATCTCGC

Supplementary Table S2. Analysis of *GhGA2ox15* promoter

Element	Sequence	Number	Function
ABRE	CACGTG	1	Cis-acting element involved in the abscisic acid responsiveness
ABRE	ACGTG	2	Cis-acting element involved in the abscisic acid responsiveness
ACE	CTAACGTATT	1	Cis-acting element involved in light responsiveness
ARE	AAACCA	2	Cis-acting regulatory element essential for the anaerobic induction
AT1-motif	AATTATTTTTATT	1	Part of a light responsive module
Box 4	ATTAAT	6	Part of a conserved DNA module involved in light responsiveness
CAT-box	GCCACT	1	Cis-acting regulatory element related to meristem expression
CCAAT-box	CAACGG	1	MYBHv1 binding site
GATA-motif	AAGGATAAGG	1	Part of a light responsive element
G-box	CACGTG	1	Cis-acting regulatory element involved in light responsiveness
G-box	TACGTG	1	Cis-acting regulatory element involved in light responsiveness
GT1-motif	GGTTAA	3	Light responsive element
