# Supplementary material for: The Gibberellin 2-Oxidase Gene GhGA2ox15 Positively Regulates Drought Resistance in Upland Cotton
Source: Int J Mol Sci. 2026 May 23;27(11):4712. doi: 10.3390/ijms27114712 (PMC13256557; doi:10.3390/ijms27114712)
Supplement: Supplementary file 1 [file ijms-27-04712-s001.zip › Supplementary Figures.pdf]

Supplementary Figures for

Gibberellin 2-oxidase Gene *GhGA2ox15* Positively Regulates Drought Resistance in Upland Cotton

Figure S1. Overexpression of *GhGA2ox15* reduces plant height in *Arabidopsis*.

Figure S2. Overexpression of *GhGA2ox15* reduces plant height in rice.

Figure S3. VIGS-mediated silencing of *GhGA2ox15* in cotton increases plant height and shoot biomass under normal growth conditions.

Figure S4. Analysis of CAT activity in leaves of control and silenced *GhGA2ox15* cotton under normal growth, drought stress and rehydration conditions.

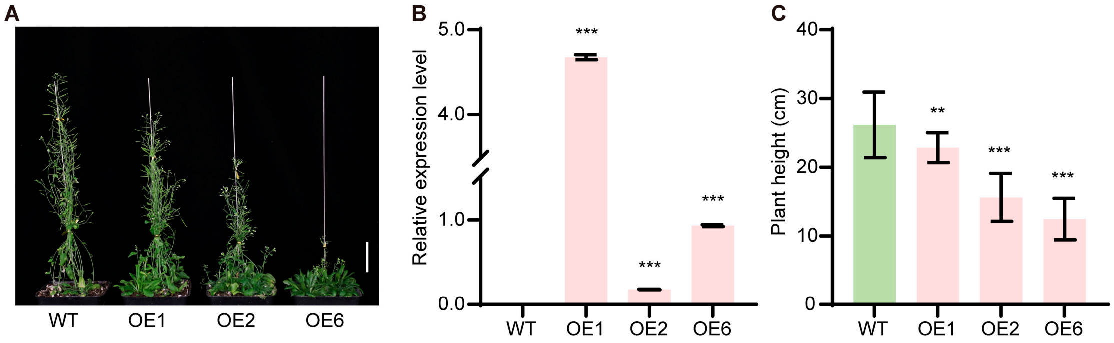

Figure S1. Overexpression of *GhGA2ox15* reduces plant height in *Arabidopsis*. (A) Phenotype of 10-week-old *Arabidopsis*. Bars = 5 cm. (B) Relative expression levels of *GhGA2ox15* in WT and T_3-generation *GhGA2ox15*-overexpressing plants determined by qRT-PCR. (C) Plant height in transgenic and WT *Arabidopsis*. Values are presented as the mean ± SE from three biological replicates. Statistical significance was determined by Student's t-test: **P* < 0.05, ***P* < 0.01, and ****P* < 0.001.

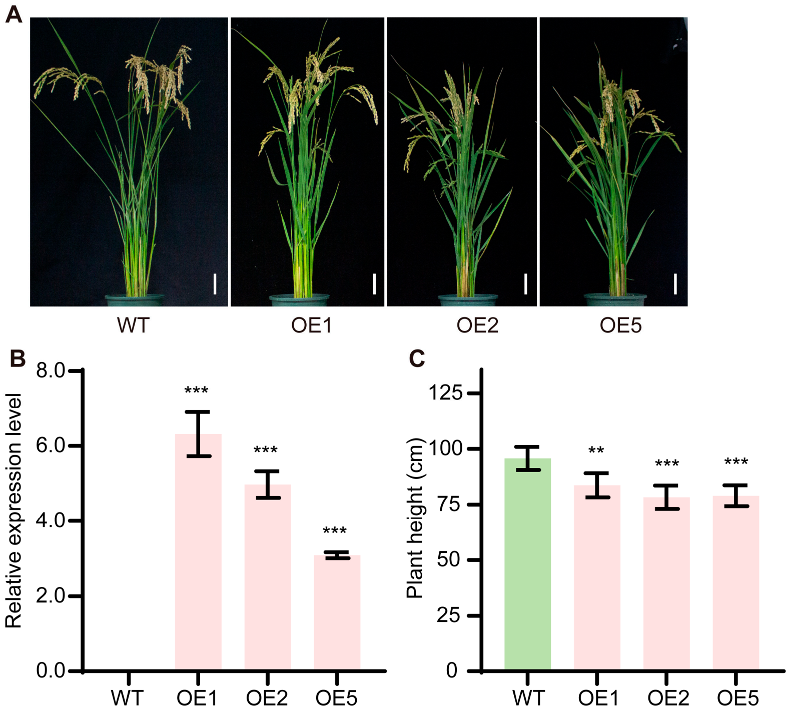

Figure S2. Overexpression of *GhGA2ox15* reduces plant height in rice (A) Morphology of WT and three independent *GhGA2ox15*-overexpressing rice lines (OE1, OE2, OE5) at the mature stage. Bar = 5 cm. (B) Relative expression levels of *GhGA2ox15* in WT and three overexpressing rice lines. Transcript levels were determined by qRT-PCR. (C) Statistical analysis of plant height in WT and *GhGA2ox15*-overexpressing rice plants. Values are presented as the mean ± SE from three biological replicates. Statistical significance was determined by Student's t-test: *$P < 0.05$, **$P < 0.01$, and ***$P < 0.001$.

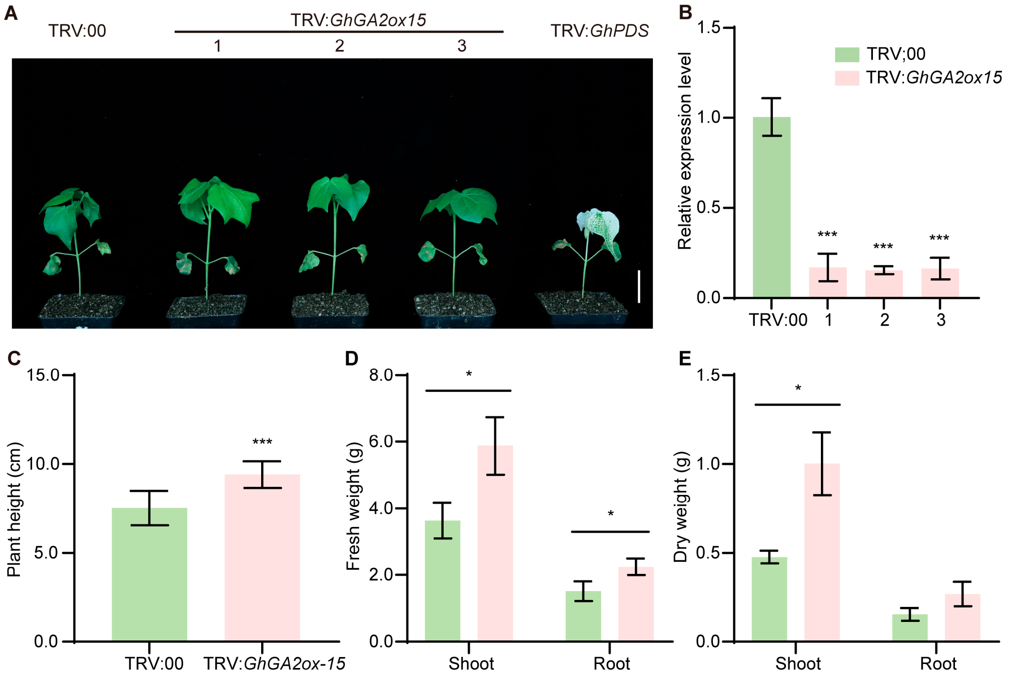

Figure S3. VIGS-mediated silencing of *GhGA2ox15* in cotton increases plant height and shoot biomass under normal growth conditions. (A) Phenotypes of control and *GhGA2ox15*-silenced plants by VIGS technology. The *GhPDS* gene was used as a positive control to verify the efficiency of VIGS in cotton. Bars = 5 cm. (B) Relative expression levels of *GhGA2ox15* in control and TRV:*GhGA2ox15* plants were detected by qRT-PCR. (C) Plant height of control and *GhGA2ox15*-silenced plants. (D-E) Fresh weight (D) and dry weight (E) of shoot and root parts of cotton. Values are presented as mean ± SE from three biological replicates. Statistical significance was determined by Student's t-test: *$P < 0.05$, **$P < 0.01$, and ***$P < 0.001$.

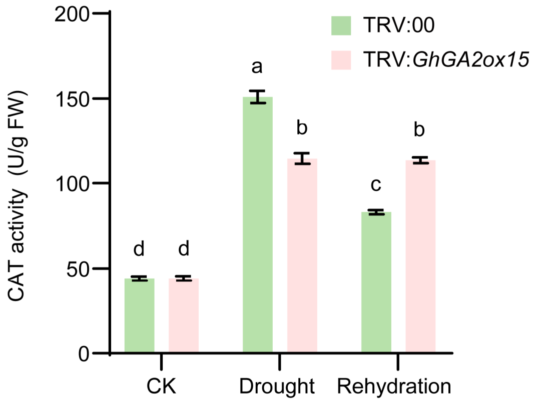

Figure S4. Analysis of CAT activity in leaves of control and silenced *GhGA2ox15* cotton under normal growth, drought stress and rehydration conditions. Values are presented as the mean ± SE from three biological replicates. Statistical significance was determined by one-way ANOVA followed by Tukey's multiple comparisons test. Different lowercase letters indicate significant differences at $P < 0.05$.
